# Supplementary material for: Empirical methods for controlling false positives and estimating confidence in ChIP-Seq peaks
Source: BMC Bioinformatics. 2008 Dec 5;9:523. doi: 10.1186/1471-2105-9-523 (PMC2628906; doi:10.1186/1471-2105-9-523)
Supplement: Additional File 1 — A variety of html documents from the USeq web site detailing the available applications, their best usage, output file type descriptions, command line menus, etc. [file 1471-2105-9-523-S1.zip › USeqUserGuides/JavaDoc.zip!/JavaDoc/trans/main/BindingPeak.html]

BindingPeak


|  |  |  |
| --- | --- | --- |
| **Overview** \| **Package** \| **Class** \| **Use** \| **Tree** \| **Deprecated** \| **Index** \| **Help** | |  |
| PREV CLASS   **NEXT CLASS** | **FRAMES**    **NO FRAMES**     **All Classes** |  |
| SUMMARY: NESTED \| FIELD \| CONSTR \| METHOD | DETAIL: FIELD \| CONSTR \| METHOD |  |


---


## trans.main Class BindingPeak

```
java.lang.Object
  trans.main.BindingPeak
```

**All Implemented Interfaces:**: java.io.Serializable

---

``` public class BindingPeak extends java.lang.Object implements java.io.Serializable ```

Holds information about a binding peak within an interval.

**See Also:**: Serialized Form

---

**Constructor Summary**

`BindingPeak(int firstPeakIndex,
int leftFlankingIndex,
int rightFlankingIndex,
int peakBP,
double score)`


| **Method Summary** | |
| --- | --- |
| `int` | `getFirstPeakIndex()` |
| `int` | `getLeftFlankingIndex()` |
| `int` | `getPeakBP()` |
| `int` | `getRightFlankingIndex()` |
| `double` | `getScore()` |
| `java.lang.String` | `toString(Oligo[] oligos, int oligoLength)`             Score, Left, Peak, Right |

**Methods inherited from class java.lang.Object**

`equals, getClass, hashCode, notify, notifyAll, toString, wait, wait, wait`

**Constructor Detail**

### BindingPeak

```
public BindingPeak(int firstPeakIndex,
                   int leftFlankingIndex,
                   int rightFlankingIndex,
                   int peakBP,
                   double score)
```


**Method Detail**

### toString

```
public java.lang.String toString(Oligo[] oligos,
                                 int oligoLength)
```

:   Score, Left, Peak, Right

---


### getFirstPeakIndex

```
public int getFirstPeakIndex()
```

---


### getLeftFlankingIndex

```
public int getLeftFlankingIndex()
```

---


### getPeakBP

```
public int getPeakBP()
```

---


### getRightFlankingIndex

```
public int getRightFlankingIndex()
```

---


### getScore

```
public double getScore()
```


---


|  |  |  |
| --- | --- | --- |
| **Overview** \| **Package** \| **Class** \| **Use** \| **Tree** \| **Deprecated** \| **Index** \| **Help** | |  |
| PREV CLASS   **NEXT CLASS** | **FRAMES**    **NO FRAMES**     **All Classes** |  |
| SUMMARY: NESTED \| FIELD \| CONSTR \| METHOD | DETAIL: FIELD \| CONSTR \| METHOD |  |


---
